# Tumor-Derived G-CSF Facilitates Neoplastic Growth through a Granulocytic Myeloid-Derived Suppressor Cell-Dependent Mechanism

**DOI:** 10.1371/journal.pone.0027690

**Published:** 2011-11-16

**Authors:** Jeremy D. Waight, Qiang Hu, Austin Miller, Song Liu, Scott I. Abrams

**Affiliations:** 1 Department of Immunology, Roswell Park Cancer Institute, Buffalo, New York, United States of America; 2 Department of Biostatistics, Roswell Park Cancer Institute, Buffalo, New York, United States of America; Roswell Park Cancer Institute, United States of America

## Abstract

Myeloid-derived suppressor cells (MDSC) are induced under diverse pathologic conditions, including neoplasia, and suppress innate and adaptive immunity. While the mechanisms by which MDSC mediate immunosuppression are well-characterized, details on how they develop remain less understood. This is complicated further by the fact that MDSC comprise multiple myeloid cell types, namely monocytes and granulocytes, reflecting diverse stages of differentiation and the proportion of these subpopulations vary among different neoplastic models. Thus, it is thought that the type and quantities of inflammatory mediators generated during neoplasia dictate the composition of the resultant MDSC response. Although much interest has been devoted to monocytic MDSC biology, a fundamental gap remains in our understanding of the derivation of granulocytic MDSC. In settings of heightened granulocytic MDSC responses, we hypothesized that inappropriate production of G-CSF is a key initiator of granulocytic MDSC accumulation. We observed abundant amounts of G-CSF *in vivo*, which correlated with robust granulocytic MDSC responses in multiple tumor models. Using G-CSF loss- and gain-of-function approaches, we demonstrated for the first time that: *1)* abrogating G-CSF production significantly diminished granulocytic MDSC accumulation and tumor growth; *2)* ectopically over-expressing G-CSF in G-CSF-negative tumors significantly augmented granulocytic MDSC accumulation and tumor growth; and *3)* treatment of naïve healthy mice with recombinant G-CSF protein elicited granulocytic-like MDSC remarkably similar to those induced under tumor-bearing conditions. Collectively, we demonstrated that tumor-derived G-CSF enhances tumor growth through granulocytic MDSC-dependent mechanisms. These findings provide us with novel insights into MDSC subset development and potentially new biomarkers or targets for cancer therapy.

## Introduction

Myeloid-derived suppressor cells (MDSC) constitute heterogeneous populations of monocytic and granulocytic-like cells reflecting various stages of differentiation that are well-regarded to play integral roles in immune suppression during diverse pathologic conditions, notably chronic inflammation, infection, trauma, graft-versus-host disease and neoplasia [1], [2], [3], [4], [5], [6], [7], [8]. Such regulatory myeloid populations accumulate in the bone marrow, blood, peripheral lymphoid tissues and sites of disease activity, such as cancer. In fact, MDSC have been identified in various human hematologic and non-hematologic malignancies, as well as in both implantable and autochthonous animal tumor models indicating that their existence represents a key component of the neoplastic process. The notion that MDSC constitute a significant barrier to effective anti-pathogen immunity has led to a comprehensive understanding of their mechanisms of immune suppression [1], [2], [3], [4], [5], [6], [7], [8], [9], [10], [11], [12].

Despite the fact that much attention has been dedicated to unraveling mechanisms by which MDSC mediate immune suppression, a larger gap remains in our understanding of the mechanisms that initiate their development. Understanding how they appear is also critical to the design of new therapeutic strategies that impede MDSC involvement in order to potentiate anti-pathogen immunity. In the case of neoplasia, it is generally thought that tumor-derived factors (TDF) govern diverse facets of MDSC biology, including their mobilization, recruitment and activation [1], [3], [4], [5], [6], [8], [13], [14], [15]. In mouse models MDSC are broadly defined as CD11b^+^Gr-1^+^ cells [1], [5], [16], [17]. More recently, MDSC have been divided into monocytic and granulocytic subsets, reflecting differential expression of the Ly6C and Ly6G epitopes. Monocytic MDSC are characterized as CD11b^+^Ly6C^high^ Ly6G^−^ or CD11b^+^Gr-1^low^ cells whereas granulocytic MDSC are defined as CD11b^+^Ly6C^low^ Ly6G^+^ cells or CD11b^+^Gr-1^high^ [6], [16], [17]. Interestingly, recent studies reported that in the vast majority of tumor models, as well as in cancer patients, granulocytic MDSC are a predominant MDSC subset [1], [17], [18], [19], [20], [21]. In fact, 70–80% of the tumor-induced MDSC response may consist of granulocytic-like cells compared to 20–30% of the cells reflecting the monocytic lineage [9], [17], [18]. It has also been reported that both subsets are equally suppressive on a per cell basis [17].

Although much interest has been dedicated to monocytic MDSC biology [3], [4], [5], [6], [13], [14], [22], less is understood about the origin of granulocytic MDSC. Therefore, in this study we focused on the mechanistic basis of granulocytic MDSC accumulation and its relevance to tumor growth. Given that the MDSC response is a consequence of altered myelopoiesis [1], [3], [4], [5], [6], [8], we reasoned that, when aberrantly expressed, tumor-derived granulocyte-colony stimulating factor (G-CSF) represents a key inflammatory component that facilitates granulocytic MDSC accumulation. Ordinarily, endogenous G-CSF regulates granulopoiesis and has an integral role in neutrophil mobilization in response to various insults [23]. Exogenous G-CSF is also important to overcome neutropenia caused by various anti-neoplastic treatments [23]. However, G-CSF exposure paradoxically can also elicit adverse effects, and inhibit innate and adaptive immunity [24], [25]; yet, the precise mechanisms by which G-CSF does so remain incompletely understood. Moreover, the idea that G-CSF may not always be beneficial to the host is supported by the findings that G-CSF is aberrantly expressed by diverse human tumors, including head and neck, cervical, ovarian, pancreatic, bladder and leukemia [26], [27], [28], [29], [30], [31].

Indeed, G-CSF production has also been shown to correlate with aberrant granulocytic or MDSC-like responses in several mouse tumor models [32], [33], [34]; however, the mechanistic link between G-CSF and granulocytic MDSC generation has not been determined. Moreover, the correlation between G-CSF and MDSC accumulation has largely focused on the global (CD11b^+^Gr-1^+^) MDSC response [35], [36], making it difficult to distinguish and conclude the impact of G-CSF on the monocytic vs. granulocytic MDSC subsets. Thus, this study focused on three fundamental questions: *1)* Does tumor-derived G-CSF drive granulocytic MDSC generation *in vivo*? *2)* Is the granulocytic MDSC response pro-tumorigenic *in vivo*? and *3)* Does recombinant G-CSF administration elicit a myeloid response phenotypically, functionally and molecularly similar to that of G-CSF-producing tumors? The latter question not only addresses the mechanistic link between G-CSF and granulocytic MDSC development *in vivo*, but also the potential implications of aphysiologic G-CSF levels in non-neoplastic disease or clinical settings.

## Results

### G-CSF is Produced at High Levels by Several Mouse Tumor Models

To test our hypothesis, we made use of two implantable orthotopic mouse models of mammary cancer that are proficient at MDSC generation, termed 4T1 and AT-3. 4T1 is a well-characterized mammary tumor model [37] that induces a rapid accumulation of systemic and intratumoral MDSC, broadly defined by their CD11b^+^Gr-1^+^ expression [1], [5], [17]. The AT-3 mammary tumor cell line, recently generated in our laboratory [38], was established from a primary mammary carcinoma of the MMTV-PyMT transgenic mouse model [39], henceforth termed MTAG mice. CD11b^+^Gr-1^+^ MDSC responses are observed under autochthonous (i.e., MTAG) and implantable (i.e., AT-3) tumor settings [38].

**Figure 1:**
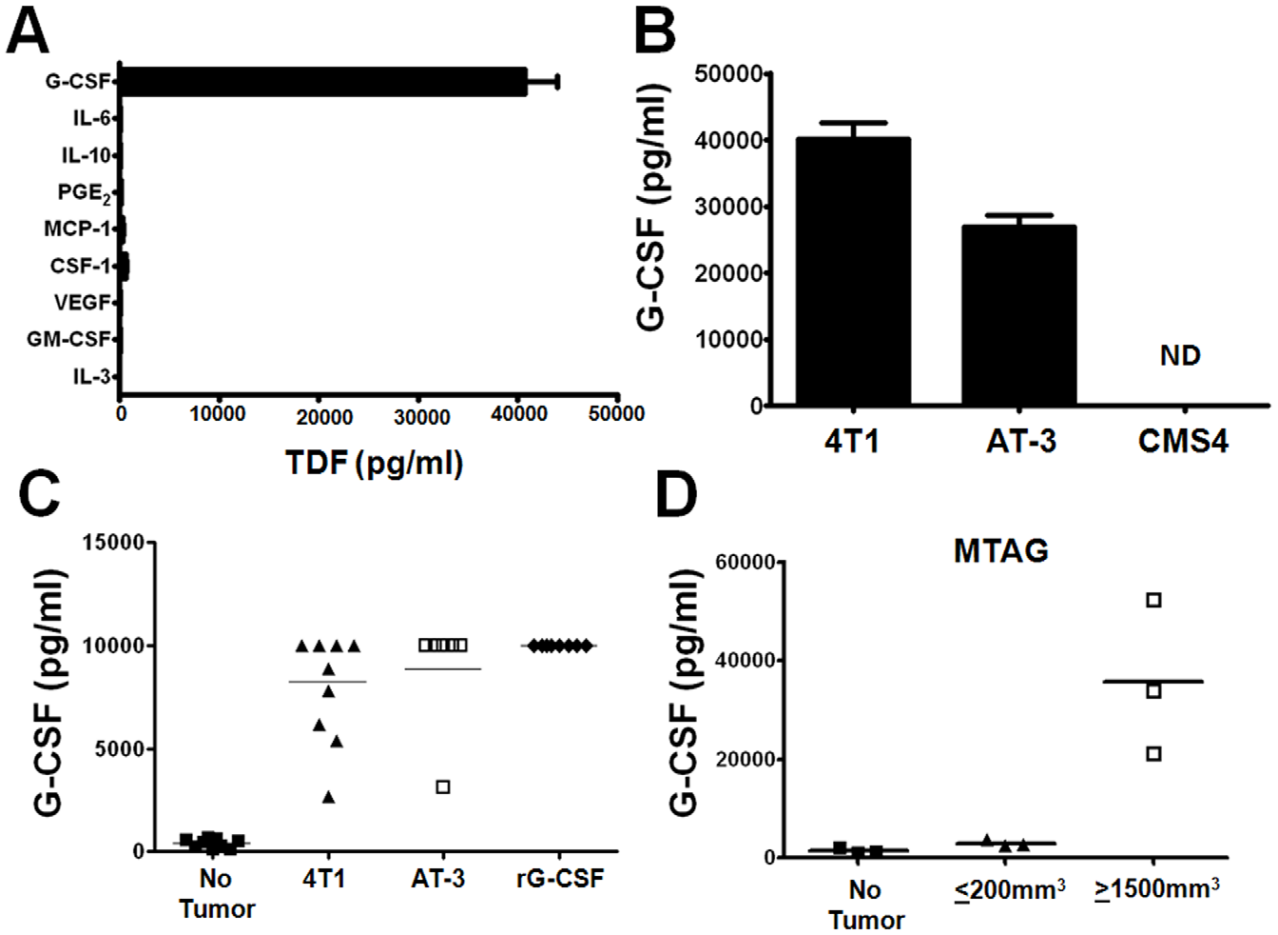
G-CSF production by various mouse tumor models. (*A*) Cell-free supernatants from 4T1 tumor cells were analyzed for the indicated TDF by ELISA. Data are reported as pg/ml/10^6^ cells/24 hr, and reflect the mean ± SEM of 3 or more separate experiments for each cytokine tested. (*B*) G-CSF levels were then analyzed in a similar fashion for the AT-3 and CMS4 tumor cell lines. Data are reported as the mean ± SEM of triplicate determinations, and are representative of 3 separate experiments. (*C*) Sera G-CSF levels determined from nontumor-bearing mice (n = 10), mice treated with recombinant G-CSF protein (10 µg daily for 5 consecutive days) (n = 8) or mice bearing 4T1 cells (n = 9) or AT-3 tumor cells pooled from two separate experiments (n = 9; overall tumor volume ≤2cm^3^). Each data point represents the results from an individual mouse. G-CSF levels of all experimental groups were significantly different compared to the nontumor-bearing control mice (*P*<0.0001). (*D*) Sera were collected from MTAG mice at the tumor burdens indicated, and analyzed for G-CSF levels. Mice bearing small tumor burdens showed significantly elevated (*P*<0.04) levels of G-CSF compared to MTAG mice without measurable tumor growth. G-CSF levels were more pronounced at larger tumor burdens (*P*<0.02, compared to ‘no tumor’ group). Each data point represents a single mouse.

We first screened tissue culture supernatants of 4T1 cells for cytokines and chemokines commonly associated with myelopoiesis and MDSC biology [1], [3], [5], [6], [8], [13], [14], [38]. We found extremely high levels of G-CSF production, whereas only marginal amounts of all other factors tested were detectable (Fig. 1A). Similarly, AT-3 cells produced high levels of G-CSF (Fig. 1B). Analysis of two additional mammary tumor cell lines, DA-3 and EMT6, yielded substantially high levels of G-CSF (Fig. S1). In contrast to these four cell lines, G-CSF production was not detectable in the CMS4 sarcoma cell line (Fig. 1B). It is worth noting that mice implanted with sarcomas generally display minimal MDSC burden [17].

We then examined G-CSF levels in the sera of 4T1 and AT-3 tumor-bearing mice (Fig. 1C). High levels of G-CSF were observed in both groups of tumor-bearing mice, whereas nominal amounts were detectable in the non-tumor-bearing control mice. As an additional control, mice were injected with recombinant G-CSF protein (Fig. 1C). Analysis of G-CSF protein revealed copious amounts in the blood reflecting a concentration range seen under tumor-bearing conditions. Lastly, sera G-CSF levels were measured in separate groups of MTAG mice at discrete stages of autochthonous tumor growth: no palpable tumors, minimal tumor burden (i.e., <200 mm^3^) and extensive tumor burden (i.e., >1500 mm^3^). Not surprisingly, G-CSF levels increased with increasing tumor burden. In contrast, little-to-no G-CSF was observed in the non-tumor-bearing controls (Fig. 1D). G-CSF levels in mice with large tumor burdens paralleled the high levels found in the implantable models. These data indicate that G-CSF is produced at high levels in multiple tumor models, and represents a potentially critical player for altered myelopoiesis and granulocytic MDSC generation. These studies also extend observations made elsewhere [32], [33], [34] regarding correlations between tumor growth and G-CSF production.

### Phenotypic and Functional Properties of CD11b^+^Gr-1^+^ cells from G-CSF-Treated Hosts

**Figure 2:**
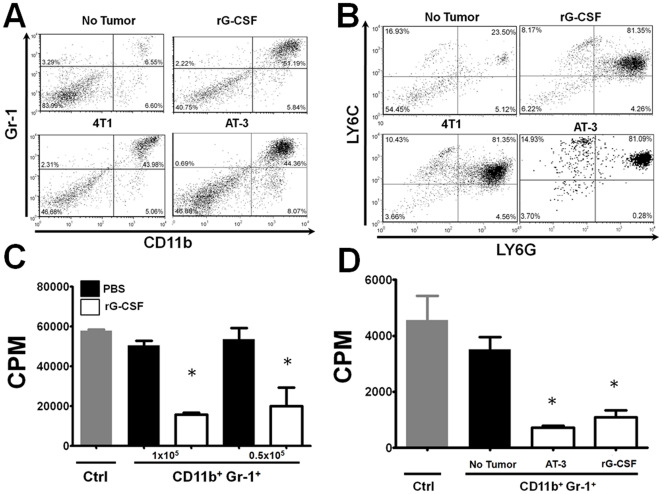
Recombinant G-CSF promotes immunosuppressive myeloid cells. (*A*) Phenotypic analysis for co-expression of CD11b and Gr-1 markers on unfractionated splenocytes from mice injected with recombinant G-CSF protein, 4T1 or AT-3 tumor cells (tumor volumes approximately 1000 mm^3^) (as in Fig. 1). Values shown in the upper right quadrant of each dot plot reveal the percentage of the CD11b^+^Gr-1^+^ myeloid fraction. Data are representative of 3 separate experiments for each group. (*B*) CD11b^+^ cells in panel *A* were gated and then re-plotted for expression of the Ly6G and Ly6C markers to determine the proportion of granulocytic (upper right) and monocytic (upper left) MDSC subsets within each group. Data are representative of 3 separate experiments for each group. (*C*) Ability of CD11b^+^Gr-1^+^ isolated from control (PBS) or G-CSF-treated mice to suppress anti-CD3 mAb-stimulated CD3^+^ T cells (1×10^5^/well) at the indicated cell densities. CD11b^+^Gr-1^+^ cells from G-CSF, but not vehicle (PBS)-treated mice significantly suppressed T cell proliferation compared to the control (i.e., no CD11b^+^Gr-1^+^ cells added; *P<0.01). (*D*) As in *C*, CD11b^+^Gr-1^+^ cells from G-CSF-treated or AT-3 tumor-bearing mice significantly (*P<0.02) suppressed allo-specific (H-2^b^ anti-H-2^d^) T cell proliferation compared to control without CD11b^+^Gr-1^+^ cells. Responders (1×10^5^/well) and stimulators (2×10^5^/well) from unfractionated splenocytes were incubated together with or without isolated CD11b^+^Gr-1^+^ cells from the indicated mice (1×10^5^/well). In both *C* & *D*, proliferation was measured by ^3^H-thymidine uptake (mean ± SEM) of triplicate determinations and are representative of two separate experiments.

To gain insights into the role of G-CSF as a potential mediator of granulocytic MDSC development, we compared the MDSC response generated in tumor-bearing mice to mice treated with recombinant mouse G-CSF protein (Fig. 2A). In control mice, the percentage of CD11b^+^Gr-1^+^ myeloid cells was <7%. In contrast, treatment of mice with G-CSF protein caused significant splenomegaly, resulting from a massive expansion of CD11b^+^Gr-1^+^ myeloid cells (Fig. 2A) with a corresponding decrease in the percentage of cells within the lymphoid compartment (Fig. S2 panel A). The accumulation of such myeloid cells was comparable to the CD11b^+^Gr-1^+^ myeloid response during AT-3 or 4T1 tumor growth (Fig. 2A).

While the co-expression of CD11b and Gr-1 markers encompass the ‘global’ MDSC population, the Gr-1 epitopes, Ly6C and Ly6G are used to delineate monocytic and granulocytic MDSC subsets [16], [17]. Using these additional phenotypic markers, splenocytes from G-CSF-treated mice displayed a prominent expansion of granulocytic-like cells (81.4% granulocytic vs. 8.2% monocytic) compared to control mice (23.5% granulocytic vs. 16.9% monocytic) (Fig. 2B). Indeed, the phenotypic pattern observed in G-CSF-treated mice paralleled that seen in AT-3 and 4T1 tumor-bearing mice (Fig. 2B). Morphologic analysis confirmed their monocytic and granulocytic lineages (Fig. S2 panel B).

Next, we examined and compared the suppressive capacity of splenic CD11b^+^Gr-1^+^ cells from control, G-CSF-treated and AT-3 tumor-bearing mice, based on inhibition of polyclonal or allo-specific T cell proliferation as readouts of suppressive behavior. Similar to what we observed previously using CD11b^+^Gr-1^+^ cells from 4T1 tumor-bearing mice [38], cells from G-CSF-treated mice also exerted significant suppression of polyclonal T cell proliferation (Fig. 2C). Moreover, we observed that CD11b^+^Gr-1^+^ cells from G-CSF-treated and AT-3 tumor-bearing mice, but not control mice significantly suppressed allo-specific T cell proliferation (Fig. 2D). Overall, the CD11b^+^Gr-1^+^ responses generated by G-CSF treatment were similar to those of 4T1 tumor-bearing mice in terms of phenotype, morphology and functional suppression.

### CD11b^+^Gr-1^high^ Cells from G-CSF-Treated and Tumor-Bearing Hosts are Also Comparable at a Molecular Level

**Figure 3:**
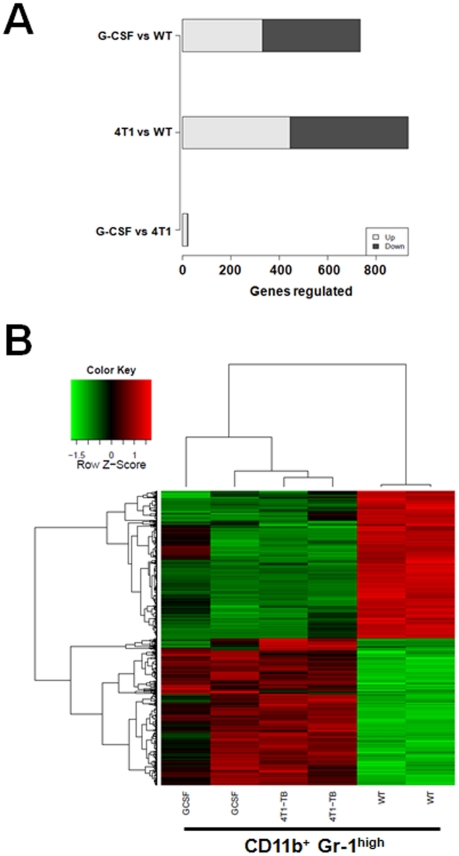
Gene expression analysis of CD11b^+^Gr-1^high^ myeloid cells from G-CSF-treated mice reveal remarkable similarities with those of 4T1 tumor-bearing mice. (*A*) Genome-wide mRNA microarray studies were conducted on purified splenic CD11b^+^Gr-1^high^ cells isolated from three groups of BALB/c mice: non-tumor-bearing control (WT), G-CSF treated (GCSF) or 4T1 tumor-bearing (TB) mice. The number of genes differentially expressed (≥2-fold change; *P*<0.01) for each of the indicated comparisons is shown in bar plot. There were 932, 734 and 22 genes showing significant expression change in 4T1-TB vs. WT, GCSF vs. WT, and 4T1-TB vs. G-CSF respectively. The fraction of genes up- or down-regulated are shown in gray or black, respectively. (*B*) Hierarchical clustering of differentially expressed (≥2-fold change; *P*<0.01) genes from comparisons for 4T1-TB vs. WT, G-CSF vs. WT, and 4T1-TB vs. G-CSF respectively. The color scale of heat map represents the relative expression level of a gene (i.e., red, increased; green, decreased) across the samples.

Global gene expression studies were performed to determine whether the granulocytic-like MDSC populations from G-CSF treated mice resembled those of tumor-bearing (TB) mice more so than those of the non-tumor-bearing control (i.e., WT) at a molecular level. To that end, splenic CD11b^+^Gr-1^high^ cell populations from WT, 4T1-TB or G-CSF-treated BALB/c mice (as in Fig. 2) were purified in two independent experiments by flow cytometry (>98% purity) and subjected to whole genome expression profiling using Illumina microarrays (see Materials and Methods). Differential expression analysis of microarray data revealed that the gene expression patterns of cells from G-CSF-treated mice resembled those of 4T1-TB hosts more than of the WT group (Fig. 3). Specifically, we identified 932 and 734 genes showing significant expression changes (i.e., >2-fold change, *P*<0.01) in 4T1-TB vs. WT comparison and G-CSF vs. WT comparison respectively, and only 22 genes were differentially expressed between 4T1-TB and G-CSF using the same significance criteria (Fig. 3A). Hierarchical clustering based on identified differentially expressed genes showed that G-CSF and 4T1-TB samples were clustered into a group separated from that of the WT samples (Fig. 3B). Overall, based on microarray gene expression profiling analysis, these data indicate that G-CSF administration can recapitulate a granulocytic-like MDSC phenotype highly comparable to that of tumor-bearing mice.

### G-CSF Blockade Diminishes Tumor Growth

**Figure 4:**
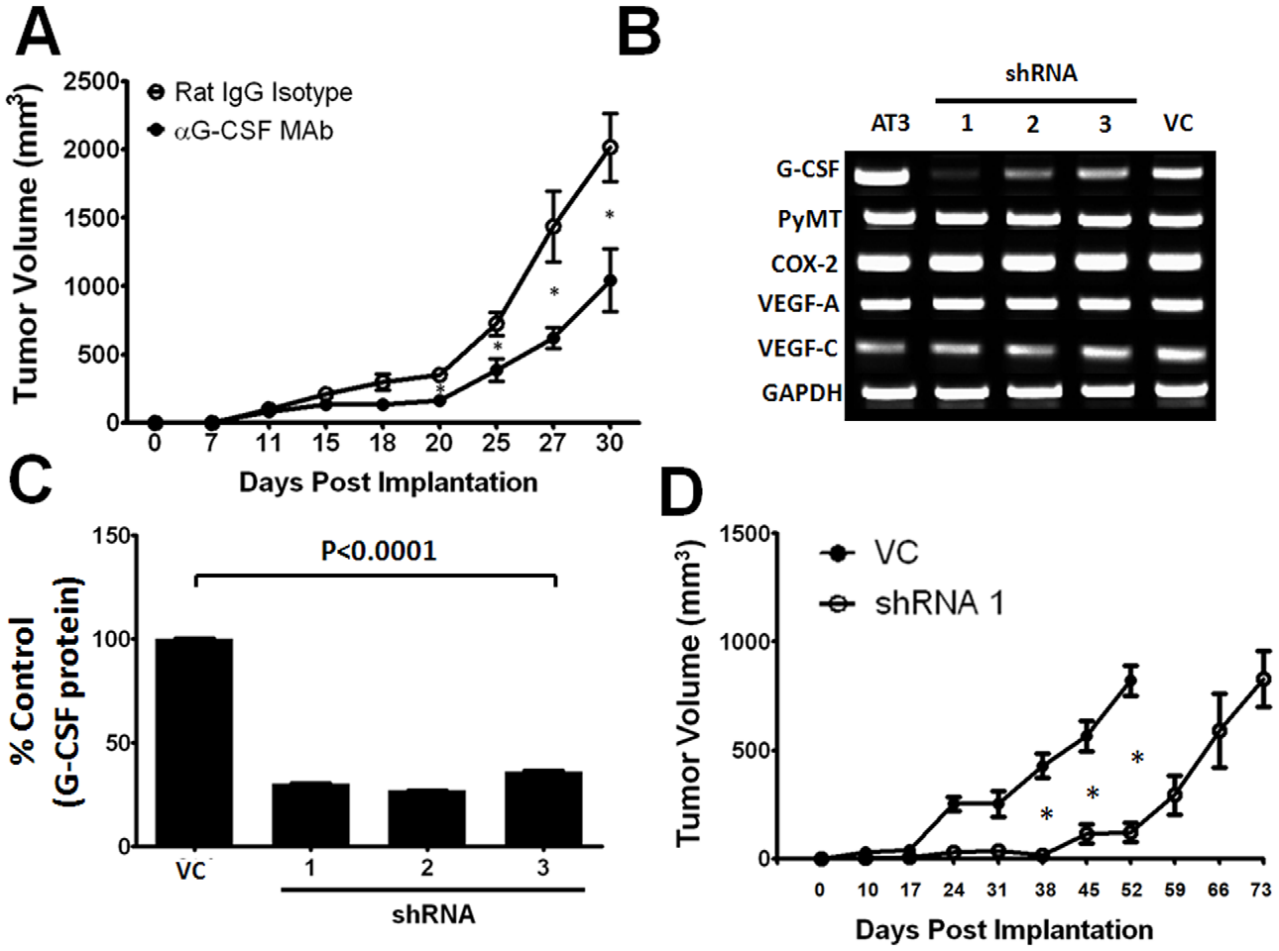
G-CSF blockade slows tumor growth. (*A*) B6 mice were implanted with AT-3 tumor cells, followed 10 days later with treatment with a neutralizing anti-G-CSF mAb or a rat isotype control (10 µg daily for 8 consecutive days). Treatment of mice with anti-G-CSF mAb significantly (*P<0.05) reduced AT-3 tumor growth at the indicated time points (from days 20-30). Data expressed as the mean ± SEM of 4 mice/group. (*B*) AT-3 cells were transduced with a scrambled shRNA sequence (non-silencing control) or different G-CSF specific shRNA sequences (constructs 1–3). Individual stable cell lines were propagated *in vitro* and analyzed for G-CSF expression, along with parental AT-3 cells, by RT-PCR for the indicated genes. (*C*) Cell-free supernatants from G-CSF knockdown vs. non-silencing control cell lines were analyzed for secreted protein by ELISA (*P*<0001)**.** (*D*) To evaluate the effect of G-CSF knockdown on tumor growth, AT-3 control and AT-3 shRNA-1 cells were implanted into separate groups of B6 mice and monitored for tumor growth (n = 5 mice each). G-CSF knockdown led to a significant delay (*P<0.03) in tumor growth at several time points indicated (from days 38 – 59). Data in panels *C & D* are reported as mean ± SEM and are representative of 3 separate experiments.

To test the hypothesis that G-CSF production is causally linked to MDSC generation and/or tumor growth, we sought to inhibit G-CSF in tumor-bearing mice using two different approaches. First, treatment with anti-G-CSF mAb significantly reduced primary tumor growth compared to mice treated with the isotype control Ab (Fig. 4A), suggesting an important pro-tumorigenic role of G-CSF during AT-3 tumor growth. It is unlikely that G-CSF acts in an autocrine fashion, as AT-3 (as well as 4T1) tumor cells do not express detectable cell surface G-CSF receptor.

Second, we made use of a loss-of-function approach, whereby G-CSF expression was down-regulated in AT-3 cells via RNA interference. Individual stable cell lines were generated using different short-hairpin (shRNA) sequences delivered via retroviral-based methods. G-CSF knockdown, relative to the non-silencing (scrambled sequence) control, was strongly observed with all constructs, as measured by RT-PCR (Fig. 4B). In contrast, genes associated with other aspects of tumor biology, such as PyMT (MTAG tumor-specific), COX-2, VEGF-A and VEGF-C remained unaffected compared to the control (Fig. 4B). G-CSF protein levels in tumor cell supernatants were also significantly reduced (Fig. 4C).

Since all three constructs appeared to be effective in silencing G-CSF expression, we arbitrarily selected cells transduced with construct 1 for further studies. Using these cells, termed AT-3 shRNA 1, we explored the impact of G-CSF loss on tumor growth and MDSC accumulation *in vivo*. To that end, syngeneic female B6 mice were implanted orthotopically with AT-3 shRNA 1 cells or the control tumor cells. Consistent with our observations using Ab-based G-CSF blockade (Fig. 4A), AT-3 shRNA 1 tumor cells grew significantly slower than control tumor cells at multiple time points (Fig. 4D). In contrast, both cell lines proliferated similarly *in vitro* (Fig. S3 panel A), suggesting that *in vivo* interactions influenced tumor growth patterns. To verify that differences in tumor growth patterns correlated with differences in G-CSF levels, sera from both groups of mice with equal tumor volumes were analyzed for G-CSF protein. We found that sera G-CSF levels were significantly lower in mice bearing AT-3 shRNA 1 tumor cells compared to the control (Fig. S3 panel B). Thus, altering tumor-derived G-CSF levels exerted a significant impact on tumor growth.

### Down-regulation of Tumor-derived G-CSF Reduces MDSC Burden

**Figure 5:**
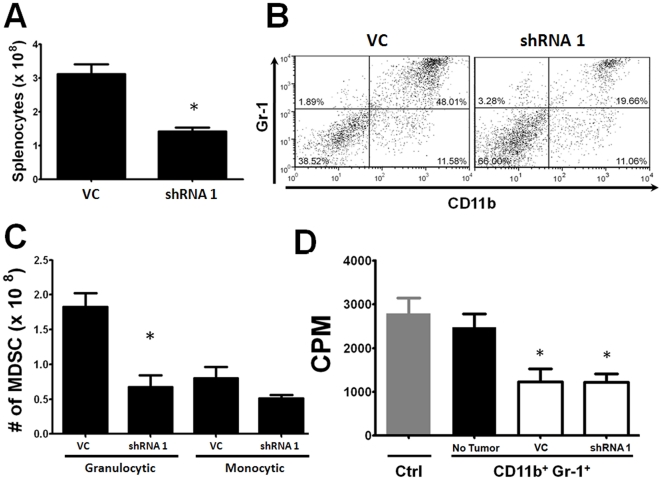
G-CSF knockdown reduces MDSC accumulation. (*A*) Splenocyte numbers were quantified from B6 mice with equivalent tumor burdens (800 – 1000 mm^3^) of vector control and shRNA-1 AT-3 cells. Splenocyte numbers were significantly higher (*P<0.004) in the control compared to the G-CSF knockdown cohort (n = 5 mice each)**.** (*B*) Flow cytometric analysis of unfractionated splenocytes in panel *A* revealed a dramatic decrease in the percentage of CD11b^+^Gr-1^+^ cells in the control compared to the G-CSF knockdown cohort (n = 5 mice each). (*C*) Splenocytes in panel *B* were evaluated for granulocytic and monocytic MDSC subsets based on additional staining with anti-Ly6G and Ly6C mAb. Absolute numbers of the MDSC subsets were then calculated using splenocyte data in panel *A*. G-CSF knockdown led to a significant decrease (*P<0.02) in the granulocytic fraction (n = 3 mice each). (*D*) CD11b^+^Gr-1^+^ cells isolated from mice in panel *A* were assayed for their ability to suppress T cell proliferation in a one-way MLR (1 of 2 separate experiments) (*P<0.02 compared to the control).

To determine whether differences in tumor-derived G-CSF levels also affected granulocytic MDSC accumulation, we analyzed splenic MDSC frequencies from both groups of mice with equal tumor volumes of 800 – 1000 mm^3^ (i.e., collected from the control group on day 52 vs shRNA 1 group on day 73). First, we observed significant reductions in splenocyte numbers in mice bearing AT-3 shRNA 1 tumor cells compared to the control (Fig. 5A). Second, to determine whether these splenic differences reflected differences in MDSC populations, we analyzed unfractionated splenocytes for co-expression of the CD11b and Gr-1 markers (Fig. 5B). AT-3 shRNA 1 tumor-bearing mice displayed a greater than two-fold reduction in the percentage of CD11b^+^Gr-1^+^ cells compared to the control (19.7% to 48.01% respectively) (Fig. 5B). Third, to delineate which MDSC subset(s) were affected most, we analyzed these preparations for granulocytic and monocytic fractions based on differential expression of the Ly6C and Ly6G epitopes in the gated CD11b^+^ fraction (Fig. 5C). Importantly, we found significant reductions in the percentage (Fig. S3 panel C) and number (Fig. 5C) of granulocytic MDSC from mice bearing AT-3 shRNA 1 tumor cells compared to the control. Conversely, the monocytic fraction remained largely unaffected, indicating a preferential effect on the granulocytic subset.

Fourth, to determine whether MDSC were affected qualitatively, splenic CD11b^+^Gr-1^+^ cells were isolated and tested for their ability to inhibit allo-specific T cell proliferation (Fig. 5D). MDSC derived from both AT-3 shRNA 1 and control tumor-bearing mice, however, displayed comparable capacity to suppress allo-specific T cell proliferation. These data indicated that G-CSF knockdown in AT-3 tumors can slow tumor growth and reduce granulocytic MDSC burden; however, the suppressive capacity of CD11b^+^Gr-1^+^ MDSC was not altered. Thus, tumor-derived G-CSF likely plays a more dominant role in affecting the *quantity* of granulocytic MDSC, which still translates to a significant effect on primary tumor growth.

### Over-expression of Tumor-Derived G-CSF Production Augments Tumor Growth

**Figure 6:**
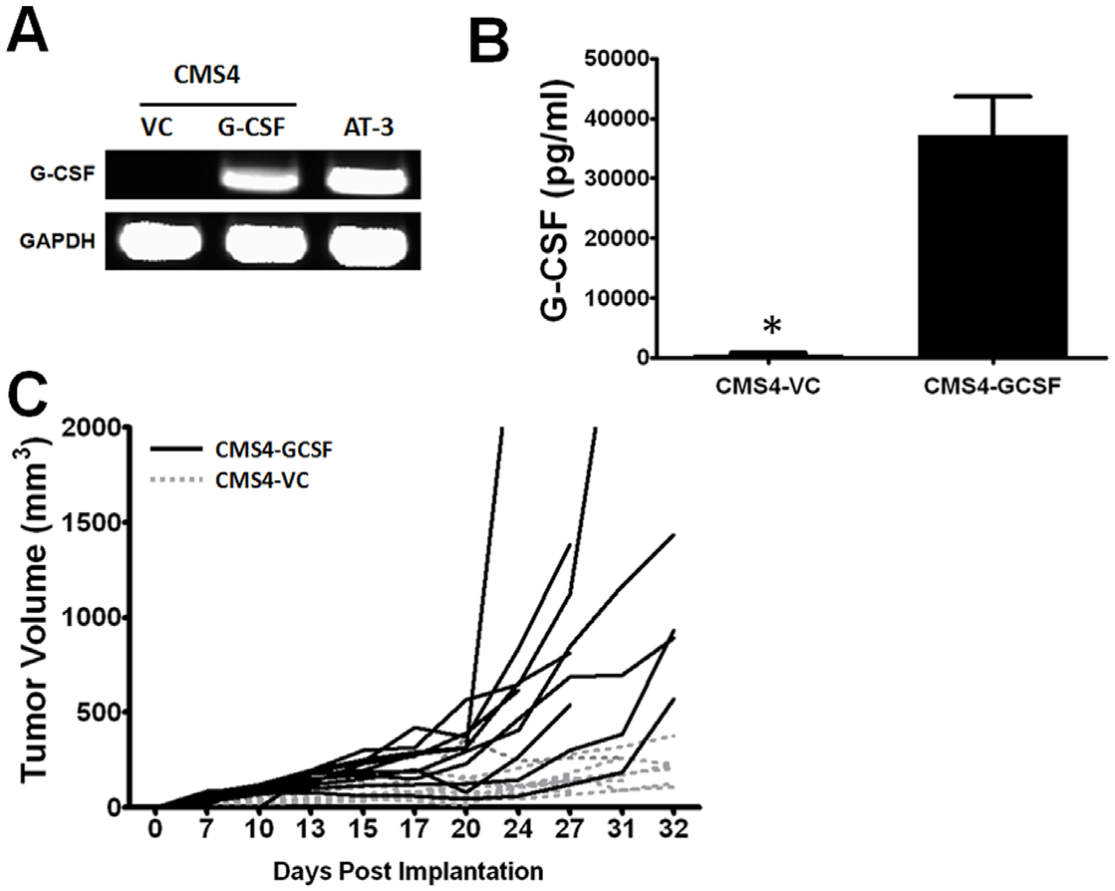
G-CSF over-expression enhances tumor growth *in vivo*. (*A*) RT-PCR analysis for G-CSF or GAPDH mRNA levels performed on parental AT-3 cells or stably transfected CMS4 cells expressing murine G-CSF or the vector control (VC). (*B*) Cell-free supernatants from CMS4 cells of panel *A* were quantified for G-CSF levels by ELISA. Data are reported as the mean ± SEM (pg/ml/10^6^ cells/24 hr) of 4 separate experiments (*P<0.0001). (*C*) BALB/c mice were implanted with either G-CSF-producing CMS4 cells or the vector control (4×10^5^/mouse) and monitored for tumor growth. In all mice, G-CSF-expressing tumors grew significantly faster than the vector control (n = 10 mice each).

To further demonstrate causality between tumor-derived G-CSF with tumor growth and MDSC accumulation, we made use of a gain-of-function approach. As shown earlier, CMS4 sarcoma cells secrete undetectable levels of G-CSF protein (Fig. 1B). Therefore, CMS4 cells were stably transfected with a murine G-CSF expression plasmid or an empty vector control. Both G-CSF-producing CMS4 cells and parental AT-3 tumor cells displayed high levels of G-CSF mRNA, whereas G-CSF transcript was undetectable in the CMS4 vector control cells (Fig. 6A). Differences in G-CSF expression were confirmed at the protein level (Fig. 6B).

In a reciprocal fashion to our G-CSF loss-of-function approach (Figs. 4 and 5), we explored the impact of G-CSF gain-of-function on CMS4 tumor growth and MDSC generation *in vivo*. Indeed, CMS4 tumors expressing G-CSF grew significantly faster than the vector control tumor cells (Fig. 6C). For reference, we observed that 10/10 mice bearing G-CSF-producing CMS4 cells displayed tumor volumes >500 mm^3^, whereas none of the control cells achieved that level at these time points. Again, this G-CSF effect was not due to intrinsic differences in tumor cell growth *in vitro*, as proliferation was comparable between both cell lines (Fig. S4 panel A). Moreover, this effect did not reflect an autocrine loop, as both groups of CMS4 tumor cells did not express the G-CSF receptor (Fig. S4 panel B). To verify that differences in tumor growth correlated with differences in G-CSF levels, sera from both groups of mice with equal tumor volumes were analyzed for G-CSF protein. We observed that sera G-CSF levels were significantly higher in mice bearing G-CSF-producing CMS4 cells compared to the control cells (Fig. S4 panel C). Thus, modulation of tumor-derived G-CSF levels exerts a significant impact on tumor growth.

### Over-expression of Tumor-Derived G-CSF Increases Granulocytic MDSC Burden

**Figure 7:**
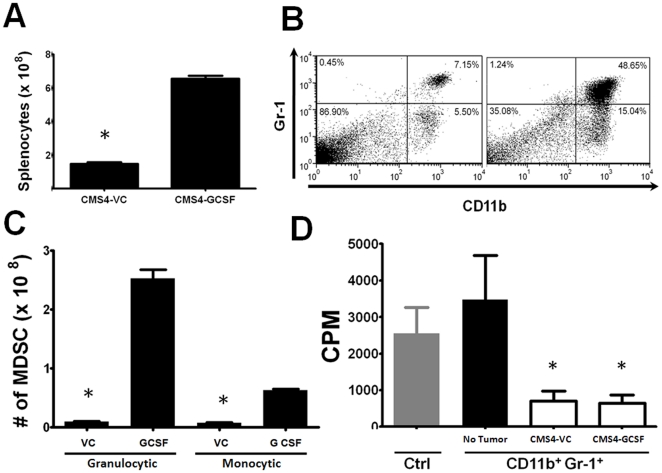
G-CSF over-expression enhances MDSC accumulation. (*A*) Splenocyte numbers were quantified from BALB/c mice with equivalent tumor burdens (∼1000 mm^3^) of vector control and G-CSF-producing CMS4 cells. This experiment is separate from that shown in Fig. 5. Splenocyte counts were significantly higher (*P<0.0002) from mice bearing the G-CSF-producing tumor cells compared to the vector control (n = 5 mice each). (*B*) Flow cytometric analysis of unfractionated splenocytes in panel *A* revealed a dramatic increase in the percentage of CD11b^+^Gr-1^+^ cells (upper right quadrant) in mice bearing the G-CSF-producing CMS4 cells compared to the vector control. (*C*) Splenocytes in panel *B* were evaluated for granulocytic and monocytic MDSC subsets based on additional staining with Ly6G and Ly6C mAb. Absolute numbers of the MDSC subsets were then calculated using splenocyte data in panel *A*. G-CSF over-expression resulted in a significant increase (*P<0.0001) in both subsets, although a more pronounced effect in terms of numbers was seen in the granulocytic subset (n = 3 mice each). (*D*) CD11b^+^Gr-1^+^ cells isolated from mice in panel *A* were assayed for their ability to suppress T cell proliferation in a one-way MLR (1 of 2 experiments) (*P<0.02 compared to the control).

Mice implanted with G-CSF-producing CMS4 cells, compared to the control, exhibited a massive increase in spleen size and splenocyte number (Fig. 7A and Fig. S4 panel D). Such analyses were performed on mice with equivalent tumor burdens of ∼1000 mm^3^. It is important to note that the control tumors required a longer time to achieve this tumor volume (i.e., 2 additional weeks). Mice bearing G-CSF-producing CMS4 cells also had an approximately 7-fold higher percentage of CD11b^+^Gr-1^+^ cells compared to those implanted with the control cells (Fig. 7B; 48.7% to 7.2%). Moreover, myeloid subset analysis revealed significant increases in the percentage (Fig. S4 panel E) and number (Fig. 7C) of granulocytic cells in mice bearing the G-CSF-producing tumor compared to the control tumor. It is also interesting to note that the number of monocytic cells also increased, although not to the same extent (Fig. 7C).

To determine whether tumor-derived G-CSF also affected function, we examined the ability of CD11b^+^Gr-1^+^ cells from control and G-CSF-producing tumor-bearing mice to inhibit allo-specific T cell proliferation. As with the AT-3 model (Fig. 5D), CD11b^+^Gr-1^+^ cells derived from both groups of tumor-bearing mice suppressed T cell proliferation at significant and comparable levels (Fig. 7D). These data indicate that MDSC are generated in this CMS4 model, and that tumor-derived G-CSF likely plays a greater role in the development and accumulation of these cells compared to affecting their suppressive behavior.

### Granulocytic MDSC Enhances Tumor Growth

**Figure 8:**
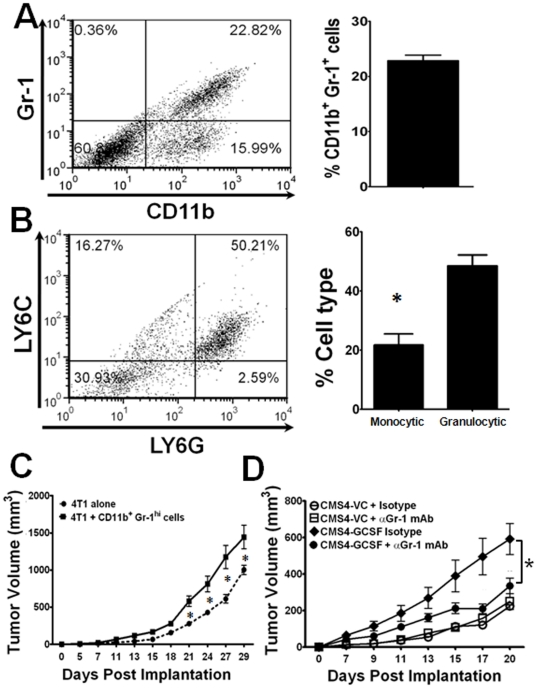
CD11b^+^Gr-1^hi^ cells from G-CSF-treated mice are pro-tumorigenic. Tumor tissue was recovered from individual 4T1 tumor-bearing BALB/c mice, enzymatically digested and then analyzed by flow cytometry for the percentages of the global MDSC response (panel *A*), as well as the granulocytic and monocytic MDSC subsets (panel *B*) as in Fig. 2. The enzyme cocktail contained DNase I (30 U/ml), collagenase A (1 mg/ml) and hyaluronidase (0.1 mg/ml, Sigma). Data are representative of 4 separate mice, and the differences between both granulocytic and monocytic MDSC subsets were significant (*P<0.003). Compilation of data points in panels *A* and *B* are shown on the right side of each dot plot. (*C*) CD11b^+^Gr-1^+^ cells were isolated from the spleens of G-CSF-treated BALB/c mice (see Fig. 2 for treatment) by two-color flow cytometric sorting. The sort focused on the recovery of CD11b^+^Gr-1^high^ cells, consistent with the granulocytic fraction. 4T1 tumor cells (5×10^4^/mouse) were then injected alone or admixed with these CD11b^+^Gr-1^high^ cells (2.5×10^4^/mouse) just prior to orthotopic tumor challenge (2∶1 ratio of tumor:myeloid population). Tumor growth data expressed as the mean ± SEM of 4 mice/group. * Indicates significant *P*-values<0.05 between the two groups at the indicated time points. (*D*) Vector control or G-CSF expressing CMS4 cells were implanted into BALB/c mice, as in Fig. 6. When tumors became palpable, all mice were treated with anti-Gr-1 mAb or rat IgG control. Data are reported as mean ± SEM of 5 mice per group (*P*<0.04 for the various comparisons at day 20).

Thus far, our data demonstrate that tumor-derived G-CSF impacts tumor growth, as well as granulocytic MDSC accumulation. However, it remained to be determined whether the effect of G-CSF on tumor growth acted through a MDSC-dependent mechanism. To do so, we adopted two approaches; one was based on a direct assessment of the functional impact of the MDSC subset in the tumor microenvironment, and the second was based on cell depletion (Fig. 8).

Phenotypic characterization of the primary tumor microenvironment, in this case the 4T1 model, revealed a substantial infiltration of CD11b^+^Gr-1^+^ cells with the vast majority bearing a granulocytic phenotype (Fig. 8A and B). Thus, it is clear that granulocytic MDSC can infiltrate or traffic to the tumor microenvironment. To determine whether such cells are also functional at the tumor site, we took advantage an ‘admix’ approach to directly assess the impact of the myeloid population on tumor growth originally described by Moses and colleagues [40], [41]. The granulocytic fraction was purified from G-CSF-treated mice by flow cytometric sorting of splenic CD11b^+^ cells co-expressing high levels of Gr-1, as in Fig. 3. Such cells were mixed with 4T1 tumor cells and then implanted into new groups of syngeneic mice. We observed that CD11b^+^Gr-1^high^ cells from G-CSF-treated mice significantly enhanced tumor growth compared to 4T1 cells injected alone (Fig. 8C). Earlier studies in this 4T1 model showed that CD11b^+^Gr-1^+^ cells from non-tumor-bearing mice are not pro-tumorigenic and do not alter tumor growth, compared to tumor cells injected alone [38]. In contrast, CD11b^+^Gr-1^+^ cells from 4T1 tumor-bearing mice enhanced tumor growth, compared to tumor cells injected alone or tumor cells admixed with myeloid counterparts from the non-tumor-bearing controls [38].

Our second approach was based on Gr-1 cell depletion, widely used in the tumor field [34], [42], [43]. Although targeting the Gr-1 populations is not necessarily MDSC-specific, it has been used as a ‘proof-of-concept’ approach to assess the potential role of Gr-1-expressing cells [34], [42], [43] in affecting a disease outcome. Besides Gr-1 cell depletion as a loss-of-function approach, to our knowledge no other alternative has been reported that can specifically deplete or inactivate MDSC without potentially impacting the tumor or host compartments. We hypothesized that if G-CSF enhances tumor growth through mobilization of granulocytic MDSC, then depletion or inactivation of such Gr-1-expressing myeloid cells would result in a reduction of tumor growth. To avert a leukopenia-induced moribund state due to repeated injections of the antibody, however, the experiment was carried out until differences in tumor growth were observed between groups. To that end, we returned to our CMS4 model in which G-CSF levels are regulated experimentally.

Treatment of mice bearing the G-CSF-expressing tumor with anti-Gr-1 mAb reduced tumor growth, which approached levels seen with the vector control tumor (Fig. 8D) (*P*<0.04 for the various comparisons at day 20). The reduction in tumor growth became more apparent over time, consistent with a time-dependent component for anti-Gr-1 mAb treatment to build up to a biologic effect. In contrast, anti-Gr-l mAb treatment had no effect on the growth of the vector control tumor compared to treatment with the isotype control (Fig. 8D). These data are consistent with the undetectable levels of tumor-derived G-CSF and low MDSC burden in the parental CMS4 model, and reinforces the likelihood that anti-Gr-1 mAb treatment did not exert non-specific effects against tumor growth. Overall, these data reveal that tumor-derived G-CSF potentiates tumor growth via granulocytic MDSC, thus unveiling a previously undescribed ‘G-CSF-granulocytic MDSC-tumor axis’ in regulating tumor progression.

## Discussion

MDSC are a major regulatory myeloid population that accumulates during a range of pathologic processes and function to inhibit innate and adaptive immunity [1], [2], [3], [4], [5], [6], [7], [8]. MDSC can be found systemically in the blood and peripheral lymphoid tissues, as well as locally at sites of disease activity. It turns out that granulocytic cells comprise a major component of the MDSC response [1], [17], [18], [19], [20], [21]; albeit, the underlying reasons for this remain unclear. Therefore, efforts to understand how they develop will have valuable implications for the design of new therapeutic paradigms. Although a number of other TDF have been linked to diverse elements of MDSC biology, namely VEGF, GM-CSF, IL-1β, IL-6, PGE_2_, IFN-γ, SCF or IL-17 [1], [3], [5], [15], [44], none have been rigorously tested to explain the connection between granulocytic MDSC response and tumor growth. Given that the granulocytic MDSC response is a likely manifestation of deregulated granulopoiesis, we hypothesized that inappropriate production of tumor-derived G-CSF contributes to granulocytic MDSC accumulation. Similarly, other groups have found positive correlations between G-CSF production and aberrant granulocytic or MDSC-like responses in several mouse models [32], [33], [34]. However, despite the correlation, the causal basis for granulocytic MDSC accumulation remained untested.

Three systematic *in vivo* approaches were taken to validate the role of G-CSF in granulocytic MDSC generation: *1)* loss-of-function using RNA interference; *2)* gain-of-function using a tumor model over-expressing G-CSF protein; and *3)* direct injection of purified G-CSF protein. Using multiple approaches, our data support the hypothesis that tumor-derived G-CSF facilitates granulocytic MDSC generation which displays both immunosuppressive and pro-tumorigenic activities. Furthermore, G-CSF protein alone was sufficient to generate granulocytic-like MDSC, which strongly recapitulated phenotypic, functional and molecular characteristics observed with tumor-induced granulocytic MDSC. These data imply that high concentrations of G-CSF acting in a systemic and aphysiologic manner can impair normal myeloid cell development or differentiation, leading to the accumulation of granulocytic-like MDSC. Thus, exogenous or endogenous stores of G-CSF may have paradoxical roles during certain pathologic circumstances, including neoplasia.

Our data do not necessarily preclude the potential role of host-derived G-CSF. However, the loss- and gain-of-function studies directly implicate a prominent role of tumor-derived G-CSF in the accumulation of granulocytic MDSC. The possibility that a ‘G-CSF-granulocytic MDSC-tumor axis’ may be important in a broader sense is supported by our data in multiple tumor models or approaches, and the fact that G-CSF production has been identified in several human cancer types [26], [27], [28], [29], [30], [31]. It is important to note that these data do not exclude the possibility that other TDF play relevant roles in granulocytic MDSC biology, especially in the absence of G-CSF production or in a G-CSF-independent manner. For example, in cases where tumors produce high amounts of GM-CSF, the monocytic fraction tends to accumulate more so than the granulocytic fraction, although the latter subset is still represented [13], [14].

In addition to its affects on granulocytic MDSC burden, we observed that tumor-derived G-CSF affects tumor growth rate. Through G-CSF blockade, as well as G-CSF loss- and gain-of-function approaches, we found that altering G-CSF levels had a direct impact on tumor growth. To directly demonstrate that granulocytic MDSC are pro-tumorigenic activity, highly purified CD11b^+^Gr-1^high^ cells from G-CSF-treated mice were collected, admixed with 4T1 cells and implanted orthotopically into naïve recipients. We found that such myeloid-tumor admixtures significantly enhanced tumor growth, demonstrating that granulocytic-like populations induced by G-CSF treatment are pro-tumorigenic within the tumor microenvironment. Moreover, depleting Gr-1^+^ cells in mice bearing G-CSF-producing, but not non-G-CSF-producing tumors facilitated a significant reduction in tumor growth, supporting a causal link between G-CSF-induced granulocytic MDSC accumulation and tumor progression.

Our data also implicate an important effect of G-CSF on the monocytic MDSC subset. Indeed, modulation of tumor G-CSF levels was accompanied by quantitative changes in the monocytic fraction, albeit, not to the same magnitude. Moreover, our data indicate that the monocytic fraction from tumor-bearing mice expressed appreciable levels of the G-CSF receptor (Fig. S5). Although it remains undetermined whether G-CSF affects the monocytic fraction directly or indirectly, this latter observation suggests that G-CSF may do so in part through a direct mechanism. Thus, the impact of tumor-derived G-CSF may have broader consequences on the overall MDSC response. Finally, it remains to be determined what regulates constitutive tumor-derived G-CSF production in the first place. As this study necessarily focused on the mechanistic basis of a ‘G-CSF-granulocytic MDSC-tumor axis’, we believe these new data now provide the framework for pursuing in detail how G-CSF is regulated by neoplastic cells.

Taken collectively, we demonstrated: *1)* a novel role of G-CSF in myeloid biology, acting at the level of granulocytic MDSC accumulation; *2)* that granulocytic MDSC can facilitate tumor growth, indicating a previously unrecognized causal link between this MDSC subset and neoplastic progression; *3)* that recombinant G-CSF administration alone to healthy mice can promote granulocytic MDSC development, which may help to explain in part the basis of certain immune-associated adverse events in healthy individuals that receive G-CSF injections to mobilize progenitor cells for use in hematopoietic transplant settings [24]; and *4)* that experimental approaches that target the initiating TDF (i.e., G-CSF) leads to tumor growth inhibition under conditions of host immunosurveillance. Current MDSC-based pharmacologic approaches include vitamin D3, retinoic acid (ATRA), KIT-specific antibody, VEGF blockade and gemcitabine (reviewed in ref. 1). Thus, therapeutic targeting of TDF that initiate granulocytic MDSC development may offer additional ways to abrogate MDSC-mediated mechanisms of tumor growth, thereby enhancing immunotherapy efficacy.

## Materials and Methods

### Mice

All experiments were conducted and approved under our Institutional Animal Care and Use Committee (IACUC) at Roswell Park Cancer Institute under protocol ID numbers 1108 M and 1117 M and in accordance with institutional regulations, NIH and Public Health Service policies. Female C57BL/6 (B6) or BALB/c (6 – 8 wks of age) mice were obtained from the NCI-Frederick Cancer Research Animal Facility (Frederick, MD). The MMTV-PyMT/B6 transgenic mouse, termed MTAG, expresses the polyomavirus middle T antigen controlled through the MMTV-LTR promoter [39], originally derived in FVB mice, was backcrossed >10 times onto a C57BL/6 (H-2^b^) background and kindly provided by S. Gendler (Mayo Clinic, Scottsdale, AZ). MTAG mice develop multifocal mammary carcinomas.

### Tumor cell lines

The 4T1 mammary tumor cell line (ATCC, Manassas, VA) was derived from BALB/c mice [37] and maintained in a RPMI-based medium [38]. The AT-3 tumor cell line was established from a primary mammary gland carcinoma of a MTAG mouse [45] and propagated in a DMEM-based medium. The CMS4 sarcoma line, a kind gift of A. DeLeo (Univ. of Pittsburgh, Pittsburgh, PA), was derived from BALB/c mice and maintained in a RPMI-based medium. *In vitro* assays were performed in a RPMI-based medium.

### *In vivo* tumor growth and G-CSF studies

Female BALB/c mice were injected orthotopically in an abdominal mammary gland with 5×10^4^ 4T1 cells. Female B6 mice were injected similarly with 5×10^5^ AT-3 cells, including those cell lines used in the knockdown studies. For the G-CSF over-expression tumor studies, syngeneic BALB/c mice were injected subcutaneously into the flank with 4×10^5^ CMS4 cells. In the admix experiments, 5×10^4^ 4T1 tumor cells were mixed with MACS-purified, MHC-matched CD11b^+^Gr-1^+^ cells at a 2∶1 ratio, respectively, prior to orthotopic implantation. Spleens, serum and tumor tissue were recovered, as appropriate. For the G-CSF blockade studies, B6 mice were injected orthotopically, and on day 7 when tumors became palpable, mice were injected intraperitoneally with 10 µg of either neutralizing anti-G-CSF mAb or rat IgG isotype (R&D Systems) for 8-consecutive days, as described [36]. For the *in vivo* depletion experiments, BALB/c mice were injected *ip* with anti-Gr-1 Ab (clone RB6-8C5; BioXcell, West Lebanon, NH) or control Ab (Rat IgG2b clone LTF-2; BioXcell, West Lebanon, NH). Antibodies were administered at 200 µg/mouse once tumors became palpable, and continued twice weekly for the duration of the experiment, similarly as described [38], [42], [43]. For all tumor experiments, mice were euthanized when tumor volumes approached 2 cm^3^ or for other health considerations. Tumor growth was measured 2 – 3 times/week in two dimensions and tumor volume was calculated using the formula (width^2^×length)/2. For recombinant G-CSF *in vivo* studies, mice were injected subcutaneously with recombinant G-CSF (10 µg/day, Peprotech, Rocky Hill, NJ) for 5 consecutive days and serum/tissues collected, similarly as described [46].

### Isolation of mouse CD11b^+^Gr-1^+^ cells

Splenic CD11b^+^Gr-1^+^ cells were purified from the indicated groups using similarly established protocols [16], [38], [47], [48]. Selections were performed using CD11b^+^ magnetic beads from Miltenyi Biotec (Auburn, CA) on an AutoMACs system. The percentage of CD11b^+^ cells was routinely >95%. Of these cells, >90% co-expressed CD11b and Gr-1 markers in both control and tumor-bearing mice, as previously reported [38].

### Flow cytometry

Myeloid preparations were pre-incubated with anti-CD16/32 mAb (BD Biosciences, San Diego, CA) to block Fc receptor binding, followed by incubation with directly conjugated primary mAb, as described [38]. Labeled cells were collected on a FACSCalibur flow cytometer (BD, San Jose, CA) and analyzed by FCS-Express software (De Novo Software, Los Angeles, CA). Antibodies reactive against the following cell surface markers were used (including appropriate isotype controls): CD11b (BioLegend, San Diego, CA), Ly6G, Ly6C, Gr-1 (BD Biosciences) and G-CSF receptor mAb (Abcam, Cambridge, MA).

### Immune suppression assays

Flat-bottomed, 96-well plates were coated with anti-CD3 mAb (1 µg/well) (BD Biosciences). Total splenic T cells were purified from naïve mice using the AutoMACS system, and plated at 5×10^4^ cells/well. Isolated CD11b^+^Gr-1^+^ cells were added to the plate at multiple CD11b^+^Gr-1^+^ cell:T cell ratios. Plates were incubated for 48 hr at 37°C, after which ^3^H-thymidine (1 µCi/well) was added for an additional 24 hr. Proliferation determined by measuring ^3^H-thymidine uptake [38]. For the mixed lymphocyte reaction (MLR)**,** one-way allogeneic H-2^b^ anti-H-2^d^ or H-2^d^ anti-H-2^b^ cultures was established. Responders (1×10^5^ unfractionated splenocytes/well from naïve mice) were cultured with irradiated (20 Gy) stimulators (2×10^5^ unfractionated splenocytes, also from naïve mice) in 96-well, U-bottomed plates. MHC-matched CD11b^+^Gr-1^+^ splenocytes were then added to the allo-assay at one or more cell densities. Plates were incubated for 96 hr at 37°C, after which ^3^H-thymidine was added for an additional 24 hr, and cells harvested as above.

### Fluorescence-activated cell sorting (FACS)

To efficiently isolate CD11b^+^ Gr-1^high^ myeloid cells from WT control, 4T1 tumor-bearing and recombinant G-CSF treated mice, we first incubated total splenocytes with CD11b (BioLegend, San Diego, CA) and Gr-1 (BD Biosciences) antibodies as indicated previously. 4T1 tumor-bearing mice had tumor burdens of >1000 mm^3^, whereas mice treated with recombinant G-CSF were given a dose regimen as previously indicated. The cells were sorted on a FACSAria cell sorter (BD Biosciences) and analyzed using FACSDiva software (BD Biosciences). The FACS resulted in >98% recovery of the CD11b^+^ Gr-1^high^ population. Total RNA was extracted from 5×10^6^ sorted cells using Qiagen's miRNeasy kit, which was quantified and subsequently used for microarray analysis.

### G-CSF under-expression and over-expression studies

RNA interference methods were used to silence G-CSF expression in AT-3 tumor cells. Stably, transduced cell lines were generated by the shRNA core facility at Roswell Park. Briefly, the retroviral vector, pSM2, was engineered to contain several independent sequences targeting murine G-CSF expression (henceforth termed, shRNA 1 (71191), 2 (68539), 3 (64790) or non-silencing/scrambled control) (Open Biosystems/Thermo Fisher Scientific, Huntsville, AL). AT-3 tumor cells were transduced with the individual G-CSF shRNA-containing retroviral supernatants. Cells were stably propagated under puromycin selection (2 µg/ml). For the G-CSF over-expression studies, murine G-CSF cDNA was PCR-cloned from AT-3 cells using the following primers: forward: 5′-AAAAAACACCATGGCTCAACTTTCTGCCC-3′; reverse: (5′-AAAAAGCGGCCGCCTAGGCCAAGTGGTGCAGA-3′. Afterwards, the gene was gel-purified, inserted into the pcDNA3.1(+) expression plasmid (Invitrogen, Carlsbad, CA), amplified, and purified under endotoxin-free conditons for use in transfection. CMS4 cells were then transfected with the expression vector or empty vector as a control using Lipofectamine (Invitrogen) per manufacturer's instructions. Cells were stably propagated under G418 (800 µg/ml) selection.

### Cytokine analysis

Cell-free supernatants from tumor cell lines (1×10^6^ cells/ml) were collected after incubation for 24 hr at 37°C. Serum was also collected, and all samples were stored at −80°C until assayed. Comprehensive analysis of cytokines was initially performed using Quansys multipex ELISA services (Quansys Biosciences, Logan, UT). Subsequently, G-CSF levels were quantified using G-CSF-specific ELISA (R&D Systems, Minneapolis, MN).

### RT-PCR analyses

Total RNA was isolated using RNeasy Mini kits (Qiagen; Valencia, CA) according to manufacturer's instructions. cDNA was synthesized using the ThermoScript RT-PCR system (Invitrogen). The cDNA was then used as the template for PCR amplification of the indicated murine genes in a PTC-200 thermal cycler (MJ Research, Waltham, MA) under the following standard conditions: 94°C for 2 min, 30 cycles (94°C for 30 sec, 60°C for 30 sec and 72°C for 1 min) and 72°C for 10 min. The following mouse primer sets were used: G-CSF forward: 5′-CTCAACTTTCTGCCCAGAGG-3′ and reverse: 5′-AGCTGGCTTAGGCACTGTGT-3′; PyMT (MTAG) forward: 5′-AGTCACTGCTACTGCACCCAG-3′ and reverse: 5′-CTCTCCTCAGTTCCTCGCTCC-3′; VEGF-A forward: 5′-CTGTGCAGGCTGCTGTAACG-3′ and reverse: 5′-GTTCCCGAAACCCTGAGGAG-3′; VEGF-C forward: 5′-TGTGGGGAAGGAGTTTGGAGC-3′ and reverse: 5′-CGGCAGGAAGTGTGATTGGC-3′; COX-2 forward: 5′-ACAACATCCCCCTCCTGCG-3′ and reverse: 5′-GCTCCTTATTTCCCTTCACACCC-3′, GAPDH forward: 5′-CATCACCATCTTCCAGGAGCG-3′ and reverse: 5′-ACGGACACATTGGGGGTAGG -3′. PCR products were separated on a 1% agarose gel and the images captured with the Chemidoc Imaging System (BioRad, Hercules, CA).

### Microarray analysis

Total RNA from highly purified splenic CD11b^+^Gr-1^high^ cells from the different experimental groups was quantified by a ND-1000 spectrophotometer (NanoDrop, Thermo Scientific) and evaluated for degradation using a 2100 Bioanalyzer (Agilent Technologies, Santa Clara, CA). Samples requirements were as follows: RIN >7, an OD 260∶280 of 1.9-2.0, and an OD 260/230 >1.8. The MouseWG-6 whole-genome gene expression array and direct hybridization assay was used for expression profiling (Illumina Inc., San Diego, CA). Initially, we converted 250–500 ng total RNA to cDNA, followed by *in vitro* transcription to generate biotin-labeled cRNA (Illumina TotalPrep RNA Amplification Kit, Ambion Inc., Austin, TX). 750 ng of the labeled probes were then mixed with hybridization reagents and hybridized overnight at 58°C to the MouseWG-6 v2 BeadChips. Following washing and staining with Cy3-streptavidin conjugate, the BeadChips were imaged using the Illumina iScan Reader to measure fluorescence intensity of each probe, where intensity corresponds to the quantity of the respective mRNA in the original sample. The expression profiles have been deposited in NCBI's Gene Expression Omnibus (GEO) with GSE accession number GSE32209. All data is MIAME compliant.

BeadChip data files were then analyzed with Illumina's GenomeStudio gene expression module and R-based Bioconductor package to determine gene expression signal levels [49]. Briefly, the raw intensity of Illumina MouseWG-6 gene expression array was scanned and extracted using BeadScan, with the data corrected by background subtraction in GenomeStudio module. The *lumi* module in the R-based *Bioconductor* Package was used to transform the expression intensity into *log2* scale [50]. The log2 transformed intensity data were normalized using Quantile normalization function. We then performed three separate comparisons for 4T1-TB versus WT, G-CSF versus WT and 4T1-TB versus G-CSF. We used the *Limma* program in the R-based *Bioconductor* package to calculate the level of gene differential expression for each comparison [51]. Briefly, a linear model was fit to the data (with cell means corresponding to the different condition and a random effect for array), and selected contrast for each comparison was performed. For each comparison, we obtained the list of differentially expressed genes (≥2-fold change) constrained by *P*<0.01. Results are reported from data collected on two biologic replicates of each group.

### Statistical analysis

For comparisons between control and experimental groups, data were recorded as mean ± SEM of the indicated number of mice or experiments. Statistical analysis was determined using a two-sided unpaired *t*-test or exact Wilcoxon Rank Sum tests, where appropriate. *P*-values less than 0.05 were considered significant.

## Supporting Information

Figure S1 **High levels of G-CSF produced by DA-3 and EMT-6 mammary carcinomas.** Cell-free supernatants from DA-3 or EMT-6 mammary carcinoma cell lines were collected and analyzed by ELISA for G-CSF secretion (pg/ml/10^6^ cells/24 hr). DA-3 cells produced high levels of G-CSF (33,000±3605 pg/ml); EMT-6 cells also produced an appreciable level of G-CSF (1,687±120 pg/ml). Data expressed as the mean ± SEM of triplicate determinations.
(TIF)

Figure S2 **Recombinant G-CSF administration results in a selective increase in CD11b^+^ Gr-1^+^ myeloid cells.** (*A*) BALB/c mice were treated with recombinant mouse G-CSF protein (10 µg/day for 8 consecutive days). Two days following the last injection, spleens were collected. Splenocytes from untreated or G-CSF-treated mice were analyzed by flow cytometry for the indicated cell surface marker. CD11b^+^Gr-1^+^ cells were strongly increased following G-CSF treatment, which was accompanied by a corresponding reduction in other major lymphoid populations. Data represent the mean percentage positive cells ± SEM (n = 5 mice/group). (*B*) Small aliquots of the indicated CD11b^+^Ly6C^high^Ly6G^-^ and CD11b^+^Ly6C^low^ Ly6G^+^ cells in Fig. 2 were collected by cell sorting, and then evaluated morphologically by a hematopathologist to verify monocytic and granulocytic cell types. Photomicrographs were taken of representative areas under 40X magnification.
(TIF)

Figure S3 **Impact of G-CSF knockdown on tumor growth** ***in vitro*** **, serum G-CSF levels and MDSC frequences** ***in vivo.*** (*A*) Control and shRNA 1 AT-3 cells were measured for potential differences in proliferation *in vitro* by the MTS assay, a variation of the MTT assay. Tumor cell lines were incubated in flat-bottomed, 96-well plate for 24 hr at multiple cell densities. After incubation time, the MTS solution was added and the extent of proliferation was determined by measuring OD at 490 nm. (*B*) Stability of G-CSF knockdown *in vivo* was determined by measuring systemic G-CSF levels from both groups of mice with comparable tumor volumes (800 – 1000 mm^3^). Data expressed as the mean ± SEM (n = 3; *P*<0.02). (*C*) Splenocytes were isolated from mice bearing control and shRNA 1 AT-3 cells at comparable tumor volumes, and analyzed for the percentages of granulocytic and monocytic subsets. G-CSF knockdown in AT-3 cells led to a significant decline in granulocytic MDSC. Data expressed as the mean positive staining ± SEM (n = 3; *P*<0.02).
(TIF)

Figure S4 **Impact of G-CSF over-expression on tumor growth** ***in vitro*** **, serum G-CSF levels and MDSC frequences** ***in vivo*** **.** (*A*) CMS4-vector control (CMS4-VC) and G-CSF-producing CMS4 cells (CMS4-GCSF) were measured for potential differences in proliferation *in vitro* as in Fig. S3. Data expressed as the mean OD (at 490 nm) ± SEM (n = 3). (*B*) Each cell line was also analyzed for G-CSF receptor expression by flow cytometry. One of 3 representative experiments is shown. (*C*) Stability of G-CSF over-expression *in vivo* was determined by measuring systemic G-CSF levels from the two groups of mice with equivalent tumor volumes (∼1000 mm^3^). Data expressed as the mean ± SEM (n = 5; *P*<0.0001). (*D*) Photograph of representative spleens from CMS4-VC or CMS4-GCSF tumor bearing mice (∼1000 mm^3^). (*E*) Splenocytes in *D* were isolated from mice bearing CMS4-VC or CMS4-G tumors, and analyzed by flow cytometry for the percentages of granulocytic and monocytic subsets, based on differential staining with anti-CD11b, Ly6C and Ly6G mAb. G-CSF over-expressing CMS4 tumors showed a significant rise in granulocytic MDSC, with a corresponding drop in monocytic MDSC relative to the control. Data expressed as the mean positive staining ± SEM (n = 5; *P*<0.0004).
(TIF)

Figure S5 **G-CSF receptor expression on monocytic and granulocytic MDSC.** G-CSF receptor expression was analyzed on splenic MDSC subsets from the indicated CMS4 tumor-bearing mice. Splenocytes were stained for CD11b and Gr-1 expression and then further gated based on differential Gr-1 levels (Gr-1^high^ or Gr-1^low^) to phenotypically distinguish granulocytic from monocytic MDSC subsets, respectively. The gated cells were then analyzed for G-CSF receptor expression relative to isotype control staining.
(TIF)
