# Supplementary figures and images for: Tumor-Derived G-CSF Facilitates Neoplastic Growth through a Granulocytic Myeloid-Derived Suppressor Cell-Dependent Mechanism
Source: PLoS One. 2011 Nov 16;6(11):e27690. doi: 10.1371/journal.pone.0027690 (PMC3218014; doi:10.1371/journal.pone.0027690)

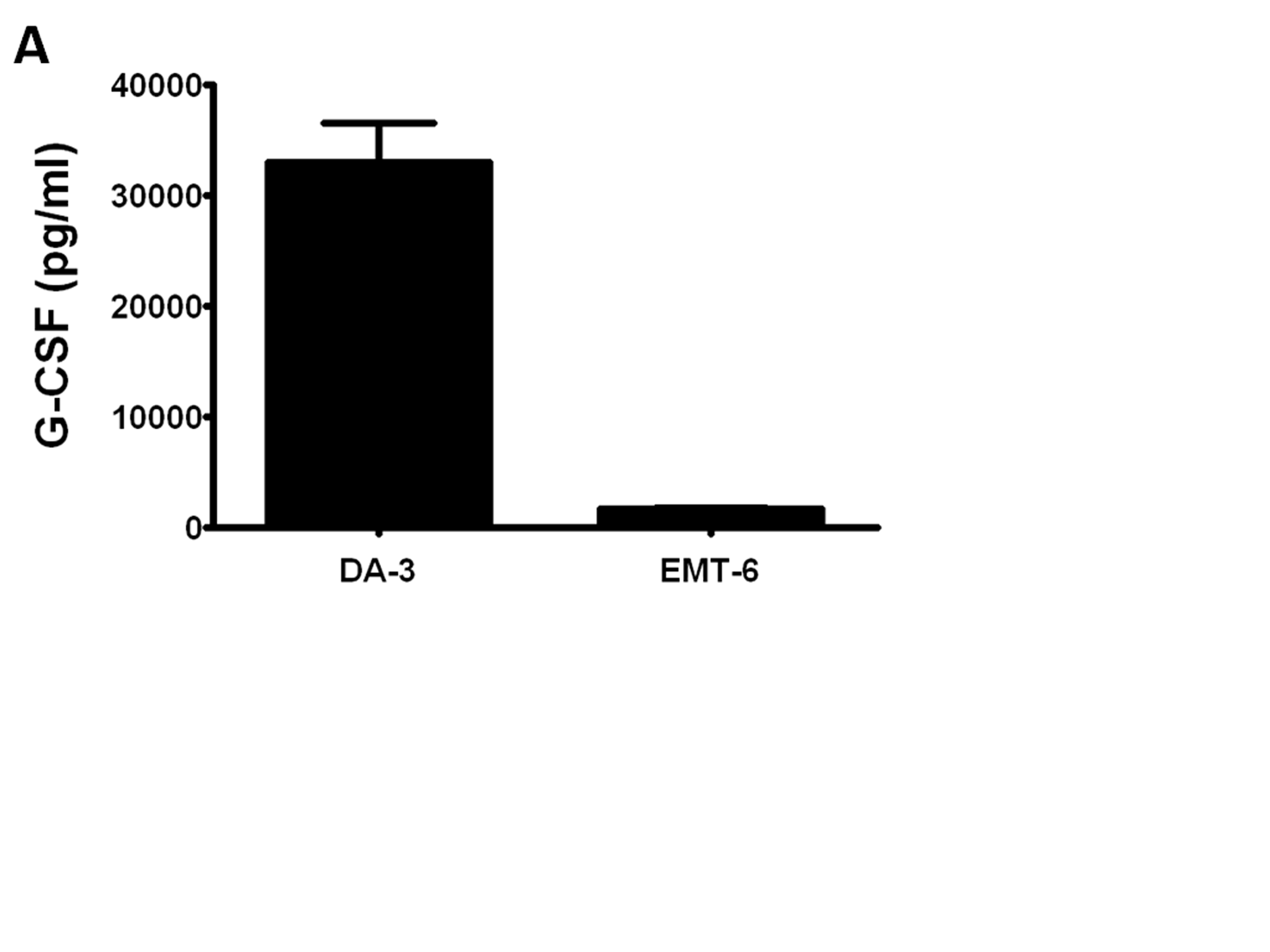

Supplement: Figure S1 — High levels of G-CSF produced by DA-3 and EMT-6 mammary carcinomas. Cell-free supernatants from DA-3 or EMT-6 mammary carcinoma cell lines were collected and analyzed by ELISA for G-CSF secretion (pg/ml/106 cells/24 hr). DA-3 cells produced high levels of G-CSF (33,000±3605 pg/ml); EMT-6 cells also produced an appreciable level of G-CSF (1,687±120 pg/ml). Data expressed as the mean ± SEM of triplicate determinations. (TIF) [file pone.0027690.s001.tif]

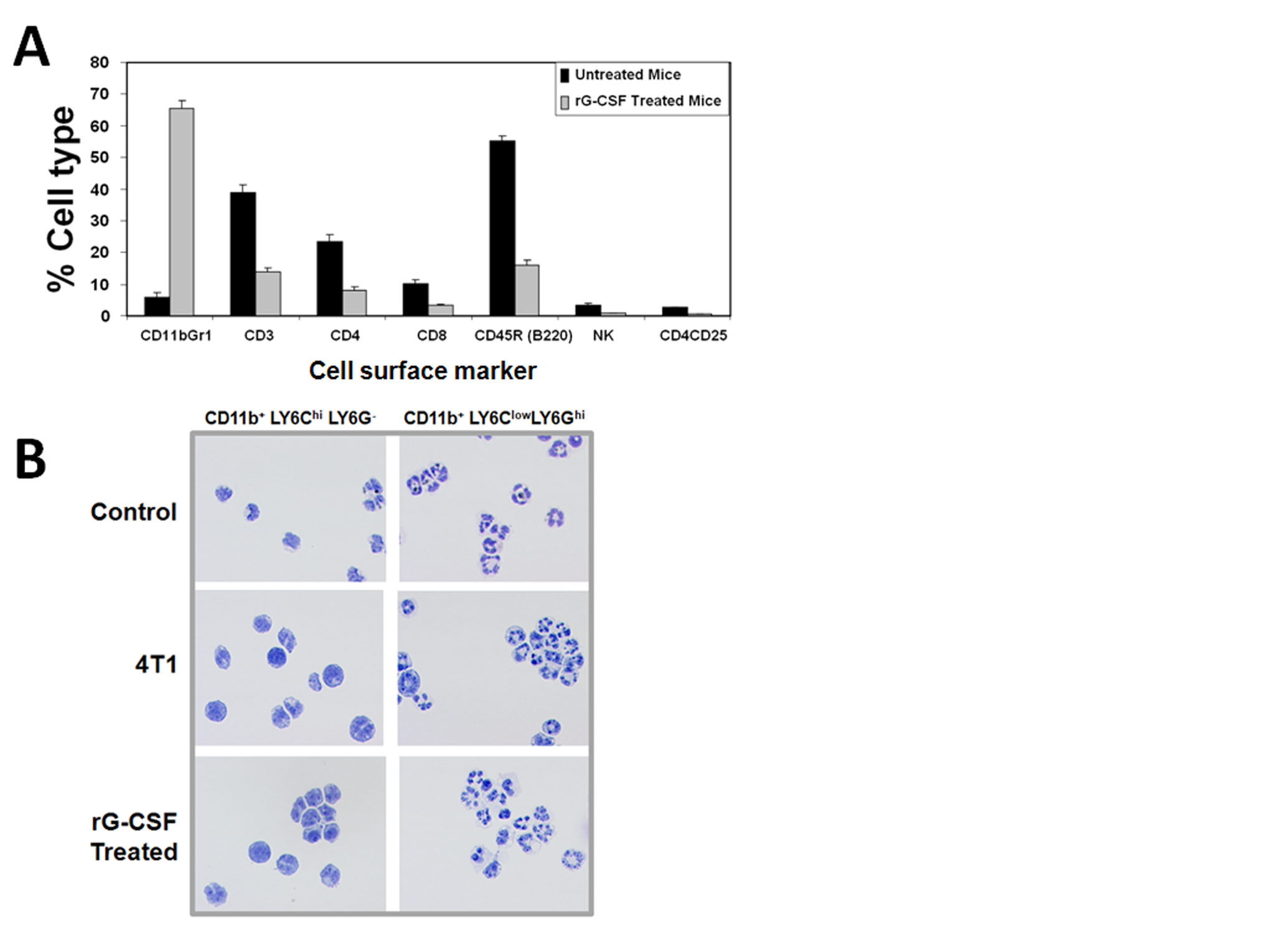

Supplement: Figure S2 — Recombinant G-CSF administration results in a selective increase in CD11b+ Gr-1+ myeloid cells. (A) BALB/c mice were treated with recombinant mouse G-CSF protein (10 µg/day for 8 consecutive days). Two days following the last injection, spleens were collected. Splenocytes from untreated or G-CSF-treated mice were analyzed by flow cytometry for the indicated cell surface marker. CD11b+Gr-1+ cells were strongly increased following G-CSF treatment, which was accompanied by a corresponding reduction in other major lymphoid populations. Data represent the mean percentage positive cells ± SEM (n = 5 mice/group). (B) Small aliquots of the indicated CD11b+Ly6ChighLy6G- and CD11b+Ly6Clow Ly6G+ cells in Fig. 2 were collected by cell sorting, and then evaluated morphologically by a hematopathologist to verify monocytic and granulocytic cell types. Photomicrographs were taken of representative areas under 40X magnification. (TIF) [file pone.0027690.s002.tif]

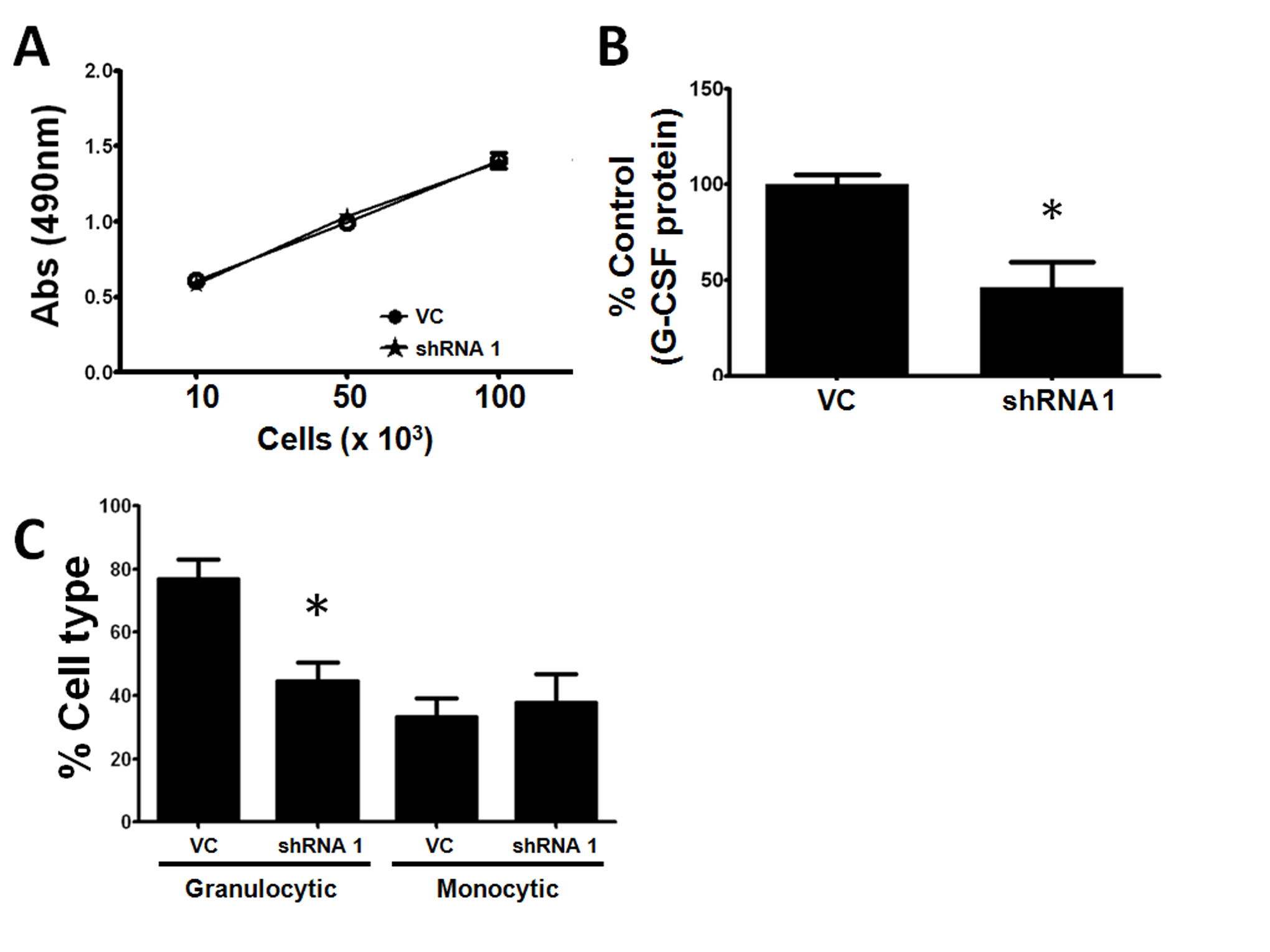

Supplement: Figure S3 — Impact of G-CSF knockdown on tumor growth in vitro , serum G-CSF levels and MDSC frequences in vivo. (A) Control and shRNA 1 AT-3 cells were measured for potential differences in proliferation in vitro by the MTS assay, a variation of the MTT assay. Tumor cell lines were incubated in flat-bottomed, 96-well plate for 24 hr at multiple cell densities. After incubation time, the MTS solution was added and the extent of proliferation was determined by measuring OD at 490 nm. (B) Stability of G-CSF knockdown in vivo was determined by measuring systemic G-CSF levels from both groups of mice with comparable tumor volumes (800 – 1000 mm3). Data expressed as the mean ± SEM (n = 3; P<0.02). (C) Splenocytes were isolated from mice bearing control and shRNA 1 AT-3 cells at comparable tumor volumes, and analyzed for the percentages of granulocytic and monocytic subsets. G-CSF knockdown in AT-3 cells led to a significant decline in granulocytic MDSC. Data expressed as the mean positive staining ± SEM (n = 3; P<0.02). (TIF) [file pone.0027690.s003.tif]

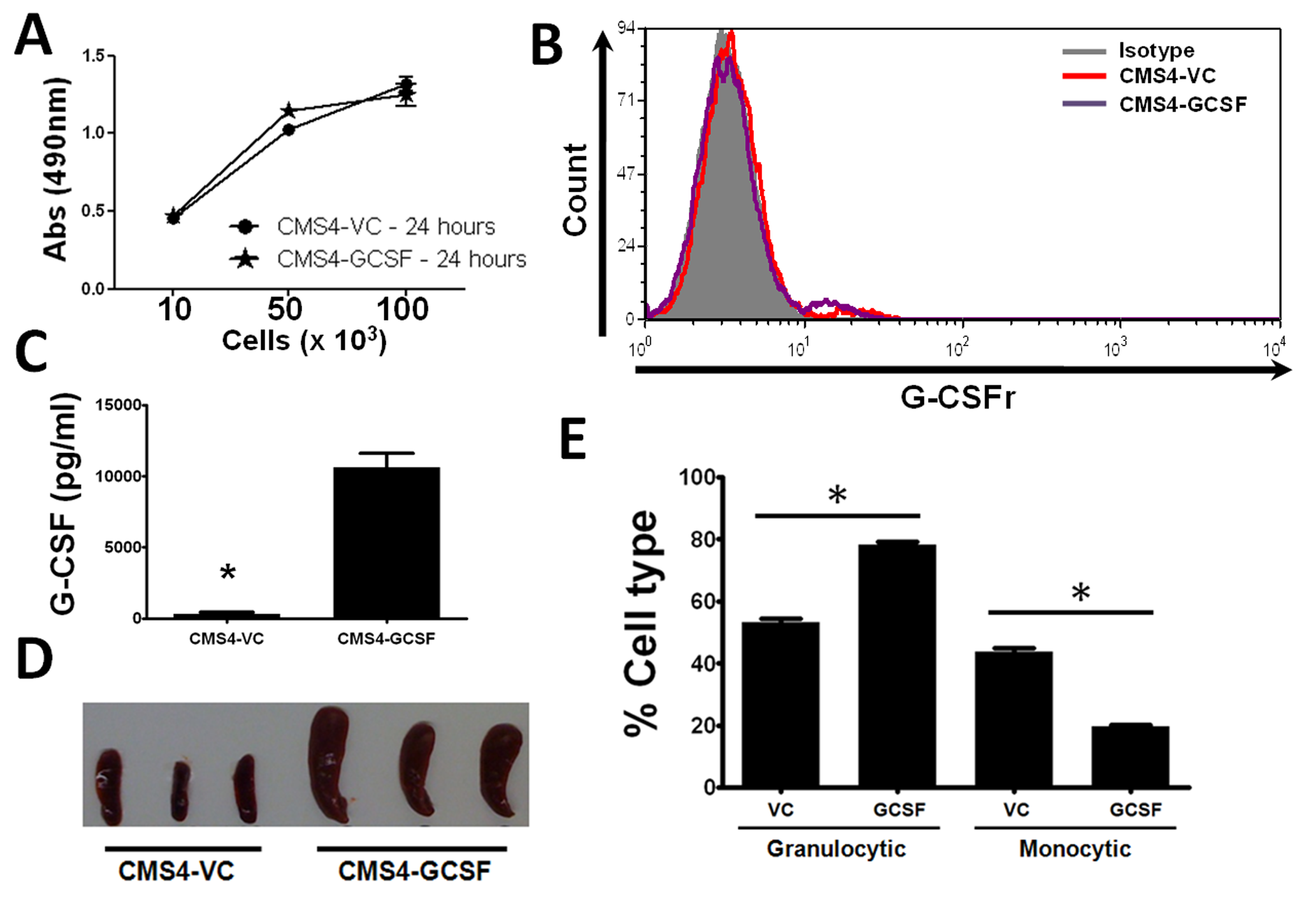

Supplement: Figure S4 — Impact of G-CSF over-expression on tumor growth in vitro , serum G-CSF levels and MDSC frequences in vivo . (A) CMS4-vector control (CMS4-VC) and G-CSF-producing CMS4 cells (CMS4-GCSF) were measured for potential differences in proliferation in vitro as in Fig. S3. Data expressed as the mean OD (at 490 nm) ± SEM (n = 3). (B) Each cell line was also analyzed for G-CSF receptor expression by flow cytometry. One of 3 representative experiments is shown. (C) Stability of G-CSF over-expression in vivo was determined by measuring systemic G-CSF levels from the two groups of mice with equivalent tumor volumes (∼1000 mm3). Data expressed as the mean ± SEM (n = 5; P<0.0001). (D) Photograph of representative spleens from CMS4-VC or CMS4-GCSF tumor bearing mice (∼1000 mm3). (E) Splenocytes in D were isolated from mice bearing CMS4-VC or CMS4-G tumors, and analyzed by flow cytometry for the percentages of granulocytic and monocytic subsets, based on differential staining with anti-CD11b, Ly6C and Ly6G mAb. G-CSF over-expressing CMS4 tumors showed a significant rise in granulocytic MDSC, with a corresponding drop in monocytic MDSC relative to the control. Data expressed as the mean positive staining ± SEM (n = 5; P<0.0004). (TIF) [file pone.0027690.s004.tif]

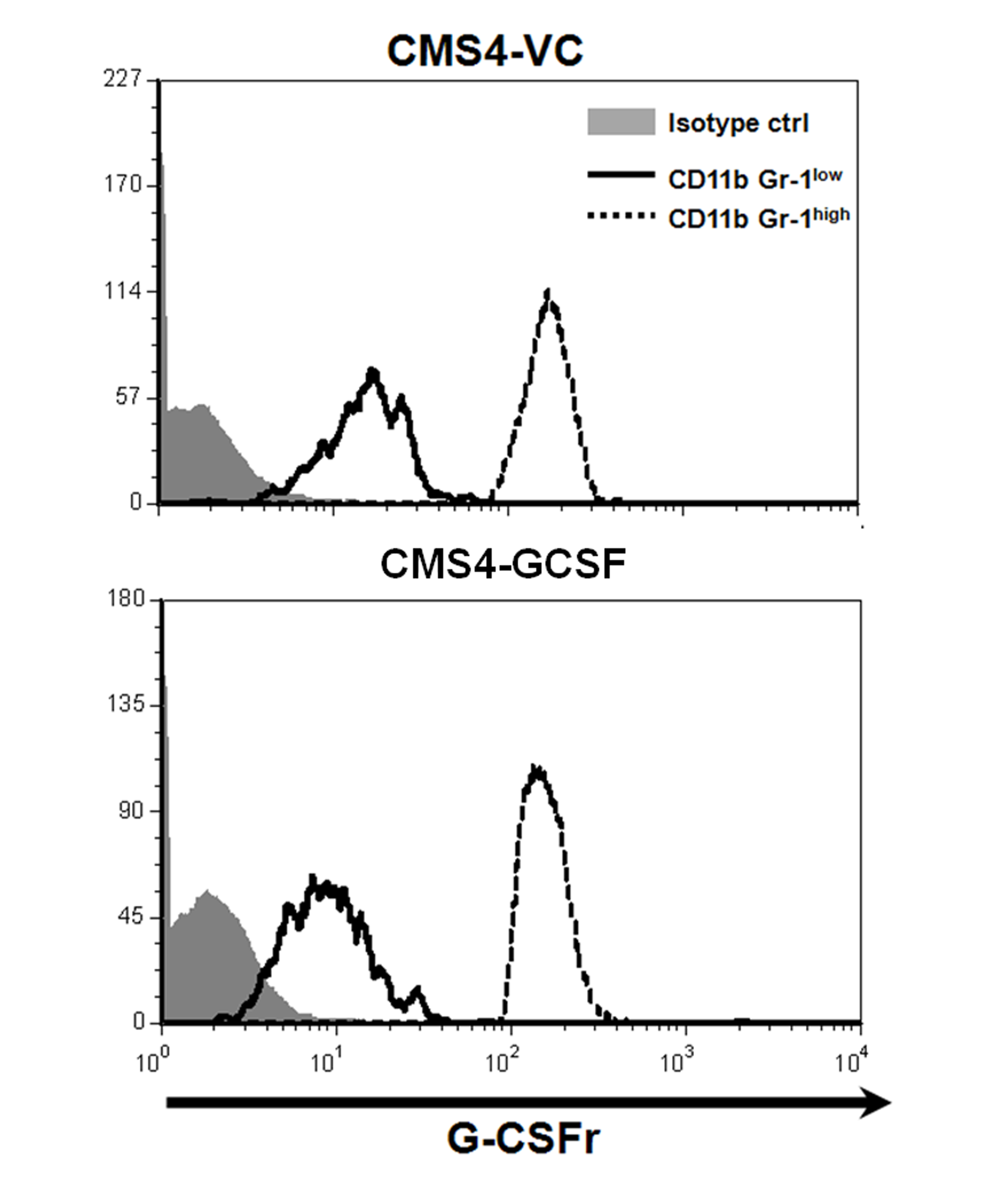

Supplement: Figure S5 — G-CSF receptor expression on monocytic and granulocytic MDSC. G-CSF receptor expression was analyzed on splenic MDSC subsets from the indicated CMS4 tumor-bearing mice. Splenocytes were stained for CD11b and Gr-1 expression and then further gated based on differential Gr-1 levels (Gr-1high or Gr-1low) to phenotypically distinguish granulocytic from monocytic MDSC subsets, respectively. The gated cells were then analyzed for G-CSF receptor expression relative to isotype control staining. (TIF) [file pone.0027690.s005.tif]
